# Inhibition of TRPV4 attenuates ferroptosis against LPS-induced ALI via Ca^2+^ pathway

**DOI:** 10.55730/1300-0152.2632

**Published:** 2022-07-26

**Authors:** Junying CAI, Guohai XU, Yue LIN, Bin ZHOU, Zhenzhong LUO, Shuchun YU, Jun LU

**Affiliations:** Department of Anesthesiology, the Second Affiliated Hospital of Nanchang University, Nanchang City, Jiangxi, China

**Keywords:** Acute lung injury, ferroptosis, transient receptor potential vanillin-4, Ca^2+^

## Abstract

Acute lung injury (ALI) is an inflammation of the lungs with high incidence rate and mortality. Ferroptosis is a new cell death, which has influence in body organs. Transient receptor potential vanillin-4 (TRPV4) channel is a key mediator of Ca^2+^, its activation induces ferroptosis. The purpose of the study is to investigate the function of TRPV4 on ferroptosis in ALI mice induced by lipopolysaccharide (LPS). In vitro, the regulation of TRPV4 on Ca^2+^ and ferroptosis was detected by CCK-8, fluorescent probe, and western blot in BEAS-2B cells. In vivo, the role of TRPV4 antagonists on ALI mice was analyzed by determination of pulmonary inflammation, pulmonary edema, and ferroptosis. In vitro, ferroptosis was induced in ALI. TRPV4 expression and intracellular Ca^2+^ concentration were up-regulated in ALI, and TRPV4 antagonist suppressed LPS-induced ferroptosis in BEAS-2B cells, including decreased MDA and ROS levels, increased GPX4 protein level and cell viability. In vivo, ALI mice showed activated ferroptosis compared with the control group, and administration of TRPV4 inhibition had protective effects on ALI mice, including improving lung pathological characteristics, and reducing the degree of pulmonary edema, inflammation, and ferroptosis. The results manifested that ferroptosis mediated lung injury in LPS-induced ALI, and TRPV4 antagonists might moderate LPS-induced damage by suppressing ferroptosis.

## 1. Introduction

Acute lung injury (ALI) is a pulmonary disease with high mortality. ALI causes severe lung diseases and induces uncontrolled pulmonary inflammation (Ferguson et al., 2012; Thompson and Matthay, 2013; Ware, 2013). The main pathological characteristics of ALI are edema, diffuse alveolar injury, and uncontrollable transference of neutrophils to the lung, leading to acute respiratory failure (Yu et al., 2021). Researchers have indicated that the lipopolysaccharide (LPS) stimulates inflammatory response and reactive oxygen species (ROS) (Wu et al., 2017; Ma et al., 2018). Moreover, the ALI model induced by LPS has been widely accepted, and it is mostly used in investigating acute respiratory distress syndrome (ARDS) (Rahman, 2002; Bae et al., 2010). Since there is currently no effective treatment for ALI, there is a need to develop potential treatments that target these factors.

In 2012, Dixon and his colleagues first reported ferroptosis (Dixon et al., 2012). Ferroptosis is characterized by the accumulation of iron-dependent lipid peroxidation to lethal levels, eventually resulting in cell death (Xu et al., 2021). It participates in various human sicknesses, and its inhibitory effect could reduce the clinical symptoms in liver injury and heart injury (Hambright et al., 2017; Fang et al., 2019; Zhou et al., 2020; Jia et al., 2021). Recently, a growing body of research has indicated that ALI has induced ferroptosis, and suppression of ferroptosis can effectively improve the disease. For instance, ferroptosis from LPS-induced ALI was reduced by reducing lipid peroxidation and increasing glutathione and glutathione peroxidase 4 (GPX4) levels (Xu et al., 2020). Studies have reported that inhibiting ferroptosis by administering drugs can reduce lung damage (Liu et al., 2020; Li et al., 2021). However, the regulatory mechanism between ferroptosis and ALI are not fully understood.

Ferroptosis is closely related to ROS accumulation. In mitochondria, Ca^2+^ signals play a crucial part in the TCA cycle and oxidative phosphorylation, of which ROS production is an important by-product (Wan et al., 1989; Feissner et al., 2009). The recent study reported that calcium oxalate induces damage to renal tubular epithelial cells by inducing ferroptosis (He et al., 2021). The transient receptor potential vanilloid-4 (TRPV4) is a Ca^2+^ ion channel, when TRPV4 channel is opened, Ca^2+^ influx and intracellular Ca^2+^ concentration increase (Ji and McCulloch, 2020). Multiple studies have shown that inhibition of TRPV4 relieves inflammation and pulmonary edema in patients with ALI (Balakrishna et al., 2014). However, it has not been reported whether TRPV4 regulates ferroptosis involved in LPS-induced ALI by regulating Ca^2+^ concentration. Thus, our aim is to explore whether inhibition of TRPV4 weakens ferroptosis against LPS-induced ALI by Ca^2+^ pathway, which provided scientific basis for the treatment of ALI patients.

## 2. Materials and methods

### 2.1. Cell culture

According to the instructions, BEGM Bronchial Epithelial Cell Growth Medium BulletKit (Zhongqiaoxinzhou Biotech, ZQ-1313) was used to culture BEAS-2B cells (Procell, China), and the cells were placed in an incubator at 37 °C with 5% CO_2_.

### 2.2. Cell viability assay

In this study, CCK-8 (Dojindo) method was utilized to detect cell viability (Zhao et al., 2016). In simple terms, BEAS-2B cells were inoculated in a 96-well plate (1 × 10^4^ cells/well) for culturing for 24 h. Next, the cells treated with LPS (10 mg/L, Sigma, Cat #: SMB00610), Fer-1 (2 μM, Ferrostatin-1, Sigma, Cat #:347174-05-4) (Liu et al., 2020), TRPV4 antagonists (10 μM, Sigma, Cat #: GSK2193874), and agonist (100 μM, Sigma, Cat #: GSK1016790A) for 16 h (Bonvini et al. 2020). Subsequently, 10 μL of CCK-8 solution was added directly to the medium and the mixture was incubated at 37 °C for 1 h. The absorbance (Abs) in each group was detected at 450 nm.

### 2.3. Western blot

The RIPA lysis buffer (Biosharp, BL504A) was used to lyse the cell samples, and the Pierce^TM^ BCA Protein Assay Kit (Thermo Scientific^™^, 23225) was used to detect the total protein concentration of different groups. Herein, the different primary antibodies used were anti-GPX4 (1:1000; Santa Crus, sc-166,570), anti-TRPV4 antibody (1:2000; Alomone Labs, ACC-034), and anti-GAPDH (1:3000; Santa Cruz, sc-47,724). The secondary antibodies used were anti-rabbit IgG (HRP-conjugated; 1:5000; Sigma-Aldrich, A-0545) and anti-mouse IgG (HRP-conjugated; 1:5000; Sigma-Aldrich, A-9044). Finally, we used the ImageJ software to quantify the protein.

### 2.4. Evaluation of malondialdehyde (MDA) level

The cells were collected, broken by ultrasonic wave, centrifuged at 8000 × *g* at 4 °C for 10 min, and the supernatant was added into a 96-well plate. Subsequently, the mixed working fluid was added to the 96-well plate. The mixture was prepared according to the Lipid Peroxidation (MDA) Assay Kit. Finally, the absorbance of each sample at 450 nm, 532 nm, and 600 nm was measured.

### 2.5. Detection of intracellular ROS

The fluorometric ROS kit (Nanjing Jiancheng, China) was utilized to test the level of intracellular ROS. In brief, DCFH-DA incubated BEAS-2B cells in the dark for 1 h and then PBS washed the cells. Subsequently, DCF fluorescence intensity was measured by flow cytometry and multiple detectors (Bio-Tek Instruments Inc.) at excitation and emission wavelengths of 485 nm and 535 nm, respectively.

### 2.6. Detection of the intracellular Ca^2+^ concentration

The cells were incubated at 37 °C for 30 min, and the supernatant was discarded. The cells were then washed with HEPES buffer saline 3 times. The next step was adding 2 mL of HEPES buffer saline. Fluorescence values were measured at the excitation wavelength of 506 nm and the emission wavelength of 526 nm.

### 2.7. Animals

C57BL/6 mice (6–8 weeks old, 20–24 g body weight) were bought from the Cloud-Clone Animal Inc. (Wuhan, China). We raised the mice in cages with food and water, and used them after quarantine and domestication for 2 weeks. All procedures involving animals were approved by the Animal Ethics Community of The Second Affiliated Hospital of Nanchang University. All operations were performed under pentobarbital sodium anesthesia to minimize the pain of the animals.

### 2.8. Animal model of LPS induced ALI

The ALI model was built by LPS injection (dissolved in sterile saline) as described (Li et al., 2019; Li et al., 2021). In the study, the mice were divided randomly into 4 groups (n = 5): the animals in the control group were injected with 0.9% NaCl (containing 0.1% DMSO), those in the ALI group were injected with LPS + 0.9% NaCl (containing 0.1% DMSO), those in the ALI + GSK2193874 group received GSK2193874 plus LPS, and those in the ALI + Fer-1 groups were injected with Fer-1 and LPS. The Fer-1 and GSK2193874 were dissolved in DMSO (Sigma-Aldrich) first and then diluted with 0.9% NaCl. The concentrations of Fer-1 (0.8 mg/kg) and GSK2193874 (10 mg/kg) were selected from Yao et al.’s (2019) and Liu et al.’s (2020) studies. Tail vein injection of Fer-1 (0.8 mg/ kg) or/and intraperitoneal injection of GSK2193874 (10 mg/kg) were performed from day 0 to day 2, once a day, three times altogether. At 1 h after the final Fer-1 and GSK2193874 injection, the mice were anesthetized with 30 mg/kg of pentobarbital sodium (Beijing Chemical, China) and then 50 μL of LPS solution (0.2 g/L) or 0.9% NaCl was administered through the trachea. After injection, the mice were placed vertically and shanked slowly for 1 min to make LPS or stroke-physiological saline solution (SPSS) evenly distributed between the left and right lungs. Twenty-four hours after LPS stimulation, the mice were euthanized by CO_2_ inhalation.

### 2.9. Lung wet/dry (W/D) weight determination

After the mice were euthanized, lung tissues were taken and immediately weighed to determine their wet weight (W). Next, the wet lung tissues were dried in an oven and the dry weights (D) were measured. Finally, the W/D ratios were calculated.

### 2.10. Hematoxylin–eosin (H&E) staining

We fixed the lungs of mice with neutral buffered formalin for 1 day. The lung tissues were dehydrated and embedded in paraffin. They were then sectioned into 3-μm thick sections by rotary microtome, and stained with H&E to analyze the pathological changes.

### 2.11. Immunohistochemistry

The expression of glutathione peroxidase 4 (GPX4) was detected by immunohistochemistry. The lung tissue sections were incubated with an anti-GPX4 antibody (1:1000; Santa Crus, sc-166,570). They were then incubated with antimouse HRP reagent (Sigma-Aldrich, A-9044). Finally, the sections were dehydrated in ethanol and removed in xylene. Light microscopy and a Nikon Photo-Imaging System (H550L, Tokyo, Japan) were used to examine the sections.

### 2.12. Statistical analysis

The GraphPad Prism 8 was used for all statistical analysis. All data were presented as the mean ± SD. The comparisons between two groups were performed using Student’s t-test, and comparisons of three or more groups were followed by one-way ANOVA followed by Tukey’s post hoc test. A p-value < 0.05 considered significant.

## 3. Results

### 3.1. Ferroptosis is rising in LPS-induced ALI

**Figure 1:**
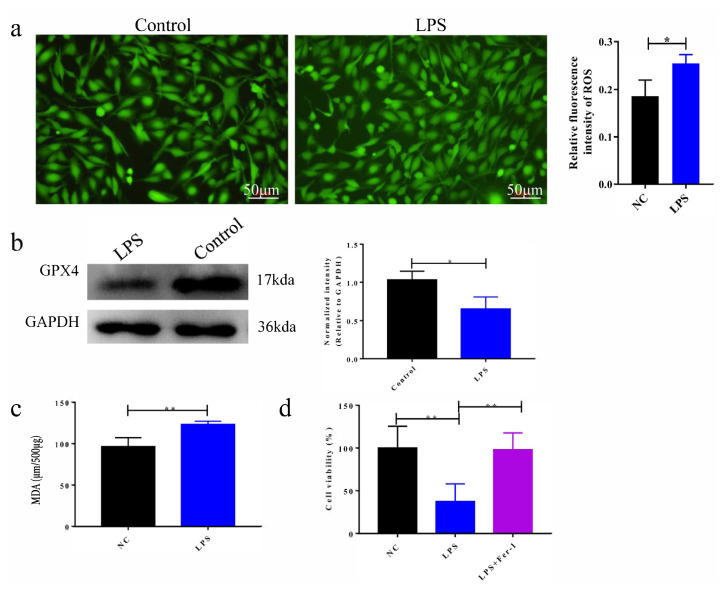
Ferroptosis increases in LPS-induced ALI. **a**. Examination of intracellular ROS by fluorometric intracellular ROS kit. **b**. Detection of GPX4 protein expression by western blot assay. **c**. Detection of MDA level by the MDA Assay Kit. **d**. Cell viability of BEAS-2B cells treated with control, LPS, and LPS + Fer-1. *p < 0.05, **p < 0.01.μ

To investigate whether ferroptosis occurs in LPS-induced ALI, BEAS-2B cells were treated with LPS and the ferroptosis level was assessed by detecting ROS and MDA levels, and GPX4 protein expression. Compared with the control group, ROS and MDA levels significantly increased, and the expression of GPX4 protein in LPS group decreased significantly (Figures 1a–1c, p < 0.05). To further confirm ferroptosis of ALI induced by LPS, we used a ferroptosis inhibitor (Fer-1) in the study. Compared with the control group, cell viability was observably reduced in the LPS group. Meanwhile, cell viability of the LPS + Fer-1 group was higher than that of the LPS group, signifying that the Fer-1 has a rescue effect on LPS-induced cell death (Figure 1d, p < 0.01). Overall, we concluded that ferroptosis is up-regulated in LPS-induced ALI.

### 3.2. TRPV4 expression and intracellular Ca^2+^ concentration are up-regulated in LPS-induced ALI

**Figure 2:**
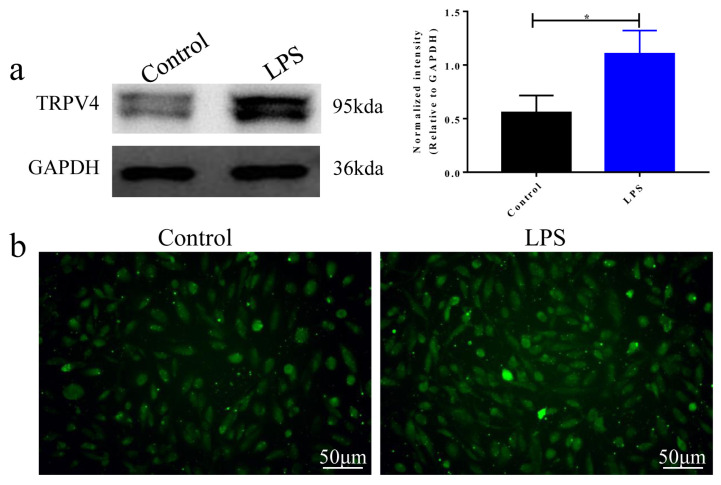
TRPV4 expression and intracellular Ca^2+^ concentration are up-regulated in LPS-induced ALI. **a**. TRPV4 protein expression detected by western blot assay. **b**. Fluorescence microscope was used to detect the intracellular Ca^2+^ concentration. *p < 0.05.

TRPV4 is a mechanosensitive Ca^2+^-permeable channel, which is required for the LPS induction of antiinflammatory/proresolution cytokines. To determine the effect of TRPV4 and intracellular Ca^2+^ concentration on BEAS-2B cell damage induced by LPS stimulation, we first examined the effects of LPS on the levels of these two indicators. Compared with the control group, LPS treatment enhanced TRPV4 protein and intracellular Ca^2+^ concentration, which suggested that LPS up-regulates TRPV4/Ca^2+^ pathway (Figures 2a and 2b, p < 0.05). These data suggested that TRPV4 protein and intracellular Ca2+ may participate in LPS-induced ALI.

### 3.3. Inhibition of TRPV4 ameliorates ferroptosis involved in LPS-induced ALI in vitro

**Figure 3:**
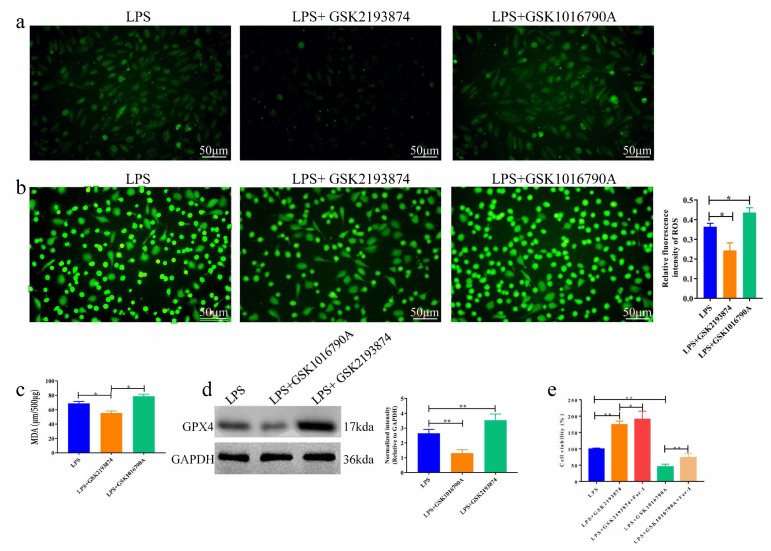
Inhibition of TRPV4 ameliorates ferroptosis involved in ALI by LPS stimulation. **a**. Confocal assay was used to detect the intracellular Ca^2+^ concentration. **b**. Examination of intracellular ROS by fluorometric intracellular ROS kit. **c**. Detection of MDA level by the MDA Assay Kit. **d**. Detection of GPX4 protein expression by western blot assay. **e**. Cell viability of BEAS-2B cells treated with LPS, LPS+GSK2193874, LPS+GSK2193874+Fer-1, LPS+GSK1016790A and LPS+GSK1016790A+Fer-1. *p < 0.05, **p < 0.01

To investigate the role of TRPV4 in LPS-induced injury of BEAS-2B cells, we treated cells with TRPV4 antagonists (GSK2193874) and/or TRPV4 agonist (GSK1016790A). We first investigated whether TRPV4 inhibitors (GSK2193874) and activators (GSK1016790A) can induce cytotoxicity in BEAS-2B cells. Compared with the LPS group, the GSK2193874 dramatically down-regulated Ca^2+^ influx, whereas the GSK1016790A had the opposite effect (Figure 3a). Furthermore, the detection of intracellular ROS by the fluorescent dye DCFH-DA showed that LPS-induced ROS levels were increased after GSK1016790A treatment, while GSK2193874 reduced ROS levels (Figure 3b). Meanwhile, the GSK2193874 significantly decreased MDA level in BEAS-2B cells, the GSK1016790A showed the opposite (Figure 3c, p < 0.05). We noted that GSK2193874 treatment induced uptake of GPX4 protein levels in BEAS-2B cells after LPS stimulation, whereas the GSK1016790A caused a decrease (Figure 3d, p < 0.01). Additionally, CCK8 experiment proved that compared with the LPS group, the cell viability was markedly reduced in the LPS + GSK1016790A group, while Fer-1 obviously relieved this effect induced by GSK1016790A (Figures 3d and 3e, p < 0.05). Contrarily, the GSK2193874 ameliorated ferroptosis induced by LPS stimulation. Collectively, these data demonstrated that inhibition of TRPV4 could ameliorate ferroptosis involved in LPS-stimulated BEAS-2B cells.

### 3.4. GSK2193874 suppresses LPS-induced ALI and ferroptosis in vivo

**Figure 4:**
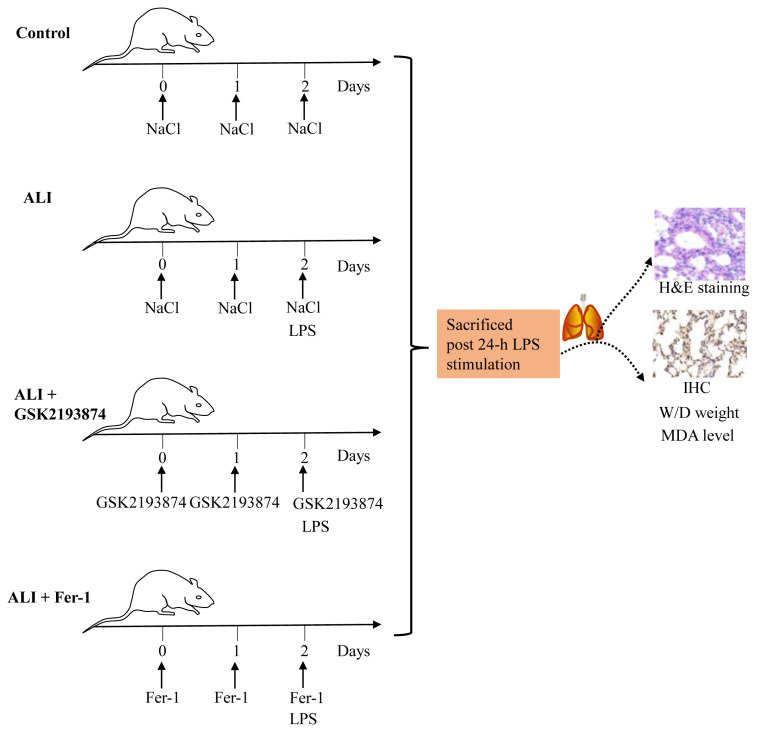
Establishment of animal model of LPS-induced acute lung injury. The male C57BL/6 mice were divided randomly into 4 groups (n = 5 per group): the control group was injected with 0.9% NaCl (containing 0.1% DMSO), the ALI group was injected with LPS and 0.9% NaCl (containing 0.1% DMSO), the ALI + GSK2193874 group was injected with GSK2193874 plus LPS, and the ALI + Fer-1 group was injected with Fer-1 and LPS.

**Figure 5:**
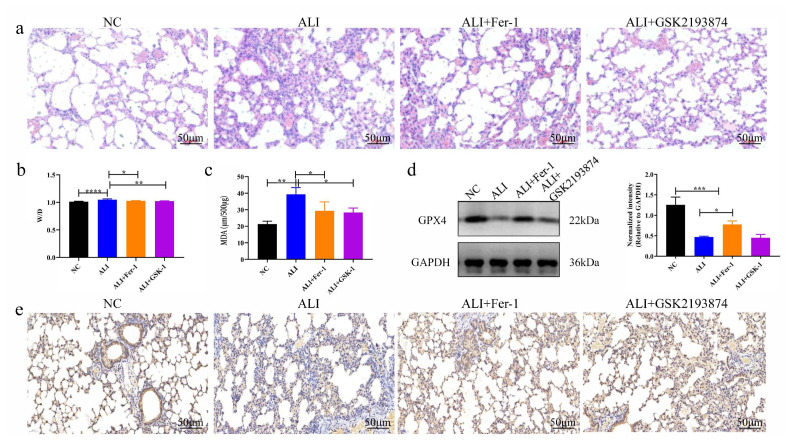
GSK2193874 inhibits LPS-induced ALI and ferroptosis in vivo. **a**. H&E staining of lung tissue sections (scale bar = 50 μm). **b**. The lung W/D weight ratio. **c**. Detection of MDA level by the MDA Assay Kit. **d**. Detection of GPX4 protein expression by western blot assay. **e**. In vivo validation of the protein expression of GPX4 using immunohistochemical staining (scale bar = 50 μm). *p < 0.05, **p < 0.01.

The ameliorating effect of TRPV4 on ferroptosis induced by ALI was further assessed in vivo. Figure 4 showed the experimental items of ALI induced by LPS. Our results manifested that there was no obvious injury in the control group (Figures 5a and 5b, p < 0.05). However, the mice in ALI groups showed the greatest degree of injury, including architecture, alveolar hemorrhage, interstitial and intraalveolar edema. Interestingly, GSK2193874 treatment significantly improved LPS-induced liver injury, as well as Fer-1 treatment. Lastly, ferroptosis was also assessed in all groups (Figures 5c–5e, p < 0.05). The ALI group had the severest ferroptosis, including the highest MDA level and the lowest level of GPX4, followed by ALI + GSK2193874 group, ALI + Fer-1 group, and the control group. Thus, these data demonstrate that TRPV4 antagonists (GSK2193874) inhibit ferroptosis involved in ALI induced by LPS in vivo.

## 4. Discussion

Although the model of LPS-induced ALI has been relatively mature, the exact mechanism of LPS-induced ALI is not fully known yet (Kolomaznik et al., 2017). Many studies have found that ferroptosis is considered a key issue in LPS-induced ALI (Liu et al., 2020). In our study, LPS-induced ALI could stimulate ferroptosis in vitro, including up-regulation of ROS and MDA levels, down-regulation of GPX4 protein expression, and the Fer-1 manifested suppression action against LPS-induced ALI. Moreover, the high prevalence disorders are usually associated with disruptions of Ca^2+^ homeostasis and TRPV4 function (Zhan and Li, 2018), such as pulmonary edema due to pulmonary venous hypertension. ALI was also observed due to pulmonary parenchymal overdistension (Hamanaka et al., 2010). However, the relationship between TRPV4 protein, Ca^2+^ concentration, and ferroptosis in LPS-induced ALI remains unclear. In our study, LPS-induced ALI showed increased TRPV4 protein expression and intracellular Ca^2+^ concentration. In brief, we found that the inhibitor of TRPV4 inhibited ferroptosis in lung epithelial cells and improved ALI.

Ferroptosis is a cell death associated with lipid peroxides. The production of excessive Fe^2+^ in cells can directly catalyze the production of lipid ROS, resulting in the continuous accumulation of lipid ROS in cells and the increase of lipid peroxidation products MDA, leading triggering ferroptosis (Dixon and Stockwell, 2014). GPX4 is an important enzyme to prevent ferroptosis (Ursini et al., 1982). So what was their relationship to ALI? The research reported that ROS up-regulate the expression of proinflammatory cytokines and adhesion molecules amplifying the tissue damage and pulmonary edema (Kellner et al., 2017), and patients with ALI had these characteristics. Other results demonstrated GPX4 may be an important intervention target in the treatment of ALI (Deng et al., 2021). Similarly, our study showed that ferroptosis was activated in LPS-induced ALI and inhibition of ferroptosis improved ALI mice. Therefore, targeted inhibition of ferroptosis may effectively inhibit ALI.

**Figure 6:**
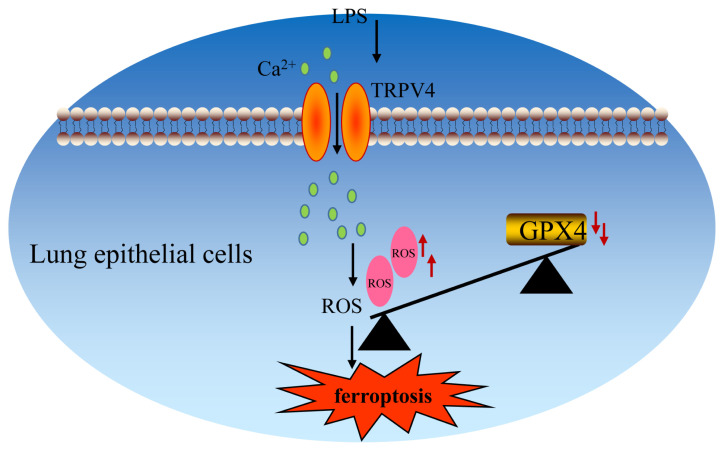
Schematic illustration of the mechanism of LPS-induced ALI resulting in ferroptosis.

TRPV4 is a nonselective Ca^2+^-permeable cation channel that is a member of the TRP channel family (Liu et al., 2019). As mentioned above, changes in their homeostasis can lead to many diseases, such as cough, bronchoconstriction, pulmonary hypertension, and ALI (Grace et al., 2017). A previous study revealed that TRPV4-dependent Ca^2+^ influx contributes to LPS-induced inflammation (Li et al., 2019). Meanwhile, mitochondria are the main sources for ROS, and their function is critically controlled by Ca^2+^ (Bertero and Maack, 2018). In our study, we found that LPS-stimulated ALI induced TRPV4 up-regulation and Ca^2+^ influx, suggesting that TRPV4 and Ca^2+^ may be involved in LPS-induced ALI. To further confirm this hypothesis, we examined the effect of TRPV4 activity on ferroptosis in ALI. We proved that inhibition of TRPV4 protein activity improved ferroptosis in ALI by LPS-stimulated in vitro, the same as other studies (Qiu et al., 2020). Besides, we observed that in vivo, breakdown of TRPV4 using GSK2193874 greatly protected the liver from injury, by reducing pulmonary edema, inflammation, and ferroptosis in LPS-induced ALI. Therefore, we suggest that the LPS-induced ALI is mainly dependent on the up-regulation of TRPV4 protein, which leads to the influx of Ca^2+^, the increase of intracellular ROS, and peroxidation product levels, and ultimately leads to changes in cell structure and function, resulting in ferroptosis (Figure 6).

## 5. Conclusion

In conclusion, the main findings of this study were as follows: (1) Ferroptosis was increased in LPS-induced ALI; (2) TRPV4 expression and Ca^2+^ influx were up-regulated in ALI stimulated by LPS; and (3) inhibition of TRPV4 improved ferroptosis in ALI stimulated by LPS in vivo or vitro. These findings suggest that ferroptosis in ALI patients was associated with TRPV4/ Ca^2+^ pathway.

## Data Availability

Data sharing is not applicable to this article as no new data were created or analyzed in this study.
